# Raised Serum Adenosine Deaminase Level in Nonobese Type 2 Diabetes Mellitus

**DOI:** 10.1155/2013/404320

**Published:** 2013-12-25

**Authors:** Vineet Kumar Khemka, Debajit Bagchi, Arindam Ghosh, Oishimaya Sen, Aritri Bir, Sasanka Chakrabarti, Anindita Banerjee

**Affiliations:** Department of Biochemistry, Institute of Post Graduate Medical Education and Research, 244 A J C Bose Road, Kolkata 700020, India

## Abstract

The role of inflammation being minimal in the pathogenesis of type 2 diabetes mellitus (T2DM) in nonobese patients; the aim of the study was to investigate the role of adenosine deaminase (ADA) and see its association with diabetes mellitus. The preliminary case control study comprised of 56 cases and 45 healthy controls which were age and sex matched. 3 mL venous blood samples were obtained from the patients as well as controls after 8–10 hours of fasting. Serum ADA and routine biochemical parameters were analyzed. Serum ADA level was found significantly higher among nonobese T2DM subjects with respect to controls (38.77 ± 14.29 versus 17.02 ± 5.74 U/L; *P* < 0.0001). Serum ADA level showed a significant positive correlation with fasting plasma glucose (*r* = 0.657; *P* < 0.0001) level among nonobese T2DM subjects, but no significant correlation was observed in controls (*r* = −0.203; *P* = 0.180). However, no correlation was observed between serum ADA level compared to BMI and HbA1c levels. Our study shows higher serum ADA, triglycerides (TG) and fasting plasma glucose (FPG) levels in nonobese T2DM patients, and a strong correlation between ADA and FPG which suggests an association between ADA and nonobese T2DM subjects.

## 1. Introduction

The incidence and prevalence of type 2 diabetes mellitus (T2DM) are increasing globally, and according to a study by World Health Organization, 300 million patients might be afflicted by the disease by 2030 with the prevalence in developing countries like India and China being estimated to cross 228 million [1, 2]. T2DM is the result of a complex interplay between various aetiological, genetic, and environmental factors [2]. A sedentary lifestyle, unhealthy food habits, and the consequent obesity related complications are responsible for the T2DM cases shooting out to epidemic proportions worldwide. However, obesity is not always linked to T2DM as ascertained by studies that indicate that the Asian T2DM phenotype is commonly less obese when defined by Body Mass Index (BMI) and around 20% of north European T2DM cases are of the nonobese type [3, 4].

Insulin resistance and impaired insulin secretion are the main physiological abnormalities associated with T2DM [4]. Immunological disturbances involving the cell mediated immune system and improper T-lymphocyte function also contribute to the pathophysiology of T2DM [5].

Adenosine deaminase (ADA) converts adenosine to inosine through an irreversible deamination reaction [6]. Highest ADA activity has been reported in lymphoid and fatty tissues, liver, skeletal muscle, and heart [7]. ADA activity measurements in the serum of obese T2DM patients have been carried out with reports indicating an increase in ADA activity in these cases. Adenosine is responsible for increasing glucose uptake into cells [8]. Thus, higher ADA activity in insulin sensitive tissue will decrease adenosine levels which in turn decrease glucose uptake into cells. ADA also plays a crucial role in lymphocyte proliferation and differentiation and is highly active in T-lymphocytes [9]. Thus, a suppression of ADA activity may help improve insulin sensitivity and inflammation, cell proliferation, and T-lymphocyte activity, all of which are putatively associated with the pathophysiology of T2DM.

Recent work has shown that a significant proportion of T2DM patients are nonobese [4]. In nonobese subjects the role of inflammation is thought to be less important because of lesser amount of adipose tissue [10]. As inflammation plays a minimal role in nonobese patients, elevated ADA levels are thought to play a crucial role compared to obese patients. As ADA has been putatively associated with inflammation, and adipose tissue inflammation is the hallmark of insulin resistance in obese T2DM patients, the serum level of ADA in nonobese T2DM is ill-defined. Our study attempted to evaluate the role of serum ADA in nonobese T2DM subjects.

## 2. Materials and Methods

### 2.1. Study Design

The preliminary case control study comprises of 56 cases and 45 healthy controls which were age and sex matched. The study was carried out in the Department of Biochemistry IPGMER, Kolkata, over a period of 6 months. The nonobese diabetic subjects were taken from the outpatient department of Endocrinology, IPGMER, while the control subjects were recruited from the subjects coming to the department for a routine health check-up. A written informed consent from the patient and control was obtained after complete explanation of the study. All the patients and controls were clinically examined and routine biochemical tests were analyzed for all subjects prior to selection. The BMI of all the T2DM subjects was measured which ranges between 19 to 24 kg/m^2^ while for control subjects it ranges between 22 and 26 kg/m^2^. The patients on insulin treatment, obesity, hypertension, ischemic heart disease, neurological disorders, renal failure, chronic liver disease, cancer, and immunological disorders were excluded from this study. The study was approved by the institutional ethics committee which follows Helsinki guidelines.

### 2.2. Biochemical Assays

3 mL venous blood samples were obtained from the patients as well as controls after 8–10 hours of fasting. All the routine biochemical parameters were analyzed by automated clinical analyzers (model Daytona, Randox). The serum ADA level was measured using a spectrophotometer based on the method by Giusti and Galanti [11]. ADA activity is described as U/L.

### 2.3. Statistical Analysis

Statistical analysis of different biochemical parameters was performed by Students' *t*-test. All variables were expressed as mean ± SD (standard deviation). The means obtained from different sample groups were compared by Kruskal-Wallis nonparametric test followed by Dunn's postcorrection test and nonparametric Mann-Whitney *U* “*t*” test. Means obtained from two normally distributed sample groups were compared by Student's unpaired two-tailed “*t*”-test. To find out the correlation between two variables, Pearson's product moment correlation coefficient was used. A value of *P* < 0.05 was considered as statistically significant. A correlation coefficient (*r*) value between −1.0 to −0.5 or 1.0 to 0.5, −0.5 to −0.3 or 0.3 to 0.5, −0.3 to −0.1 or 0.1 to 0.3, and −0.1 to 0.1 was considered as strong, moderate, weak and no correlation respectively (http://explorable.com/statistical-correlation). All statistical analyses were performed by using Graph Pad prism software (version 5, 2007, San Diego, California, USA). Statistical analysis for sex distributions was evaluated by chi-square test by using statistical software STATA (version 8, Copyright 1984–2003, Stata Corporation, Texas, USA).

## 3. Results

The demographic and biochemical profile of the nonobese T2DM subjects and healthy controls is presented in Table 1. There was no significant difference in age and sex distribution in either of the two groups while BMI showed a statistical significance between nonobese T2DM and control subjects (Table 1). Fasting plasma glucose, HbA1c and serum cholesterol, and serum triglyceride levels were elevated while serum HDL levels were lower in nonobese T2DM subjects compared to healthy controls which were found statistically significant (Table 1).

**Figure 1:**
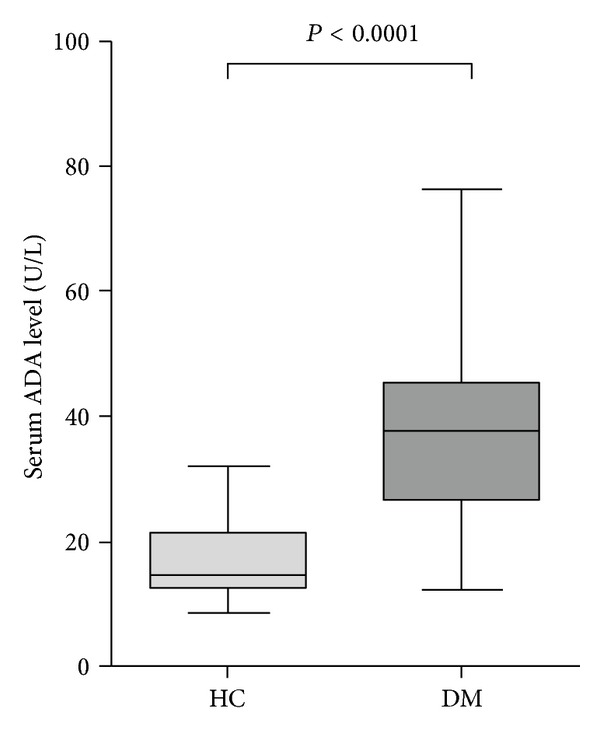
Serum adenosine deaminase levels among healthy control and nonobese T2DM subjects. Box and Whisker plots represent median, upper median, lower median, and minimum to maximum range of serum adenosine deaminase levels (ADA) among control and T2DM subjects. Values are expressed as the means ± SD. Statistically significant difference, *P* < 0.0001, control versus T2DM.

**Figure 2:**
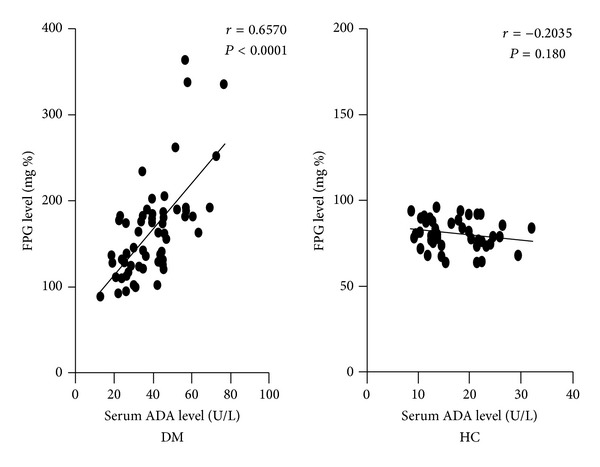
*XY* scatter plots between fasting plasma glucose (FPG) levels and serum levels of adenosine deaminase (ADA) in nonobese T2DM subjects and healthy controls (HC). Correlation coefficient (*r*) represents the degree and nature of correlation between the FPG levels and serum ADA levels in nonobese T2DM subjects and HC as described in Section 2.3. A value of *P* < 0.05 was considered statistically significant.

Serum ADA level was found significantly higher among nonobese T2DM subjects with respect to controls (38.77 ± 14.29 versus 17.02 ± 5.74 U/L; *P* < 0.0001) (Figure 1). As presented in Figure 2, serum ADA level showed a significant positive correlation with fasting plasma glucose (*r* = 0.657; *P* < 0.0001) level among nonobese T2DM subjects, but no significant correlation was observed in controls (*r* = −0.203; *P* = 0.180). However, no correlation was observed between serum ADA level compared to BMI and HbA1c levels.

## 4. Discussion

Being a multifactorial disease, T2DM is characterised by deranged protein and fat and carbohydrate metabolism secondary to insulin resistance. Early identification of insulin resistance helps in minimizing the further associated complications. Due to various complexities in the methods for determining insulin resistance, they are not commonly used for all patients. This study intends to evaluate the serum level of ADA in the pathogenesis of insulin resistance in nonobese T2DM patients.

ADA distribution varies between different tissues, but highest concentration occurs in lymphoid and fatty tissues [7]. Adenosine is involved in insulin mediated glucose uptake in skeletal muscle and high ADA activity tends to decrease glucose uptake into cells and thus contributes to insulin resistance [8]. It has been shown in some *in vivo* and *in vitro* studies that adenosine increases gluconeogenesis and glycogenolysis and stimulates glucose formation [12, 13]. It also interacts with A1 and A2 adenosine receptors modulating myocardial functions [14].

DPP-4 is an enzyme that acts as an important immune regulator by interacting with CD3 and acting as a costimulator for CD4+ T cells. It also regulates glucose homeostasis by hydrolysing integrins. DPP-4 binds ADA with high affinity and as adenosine causes apoptosis and inhibits differentiation of T lymphocytes by activating P1 adenosine receptors, interaction of ADA with DPP-4 can lead to T cell proliferation and increased cytokine production which can interfere with insulin signalling [15–18]. Moreover, ADA plays an important role in lymphocyte maturation and activity, whose deficiency is associated putatively with impaired immune function. Thus, suppression of ADA activity may help improve insulin sensitivity and inflammation, cell proliferation, and T-lymphocyte activity, all of which are associated with the pathophysiology of T2DM. Several reports also suggest that ADA modulates insulin action [19].

Adipose tissue inflammation is considered as a key event in the pathogenesis of insulin resistance [10] and obesity causes increased macrophage accumulation within adipose tissue [20]. Increased lipolysis in white adipose tissue releases free fatty acids which in turn impairs insulin signalling [21–24].

Elevated ADA levels have been reported in T2DM [25] and ADA activity correlated with glycemic control in T2DM patients [18, 25]. Further, metformin, which decreases insulin resistance also lowers ADA levels [14], thereby establishing positive correlation between ADA and insulin resistance. Previous works have shown that ADA levels are elevated in T2DM compared to controls [5] and ADA levels are positively correlated with fasting serum glucose, insulin, and HOMA [14]. Our study shows that serum ADA level is higher in nonobese T2DM subjects as compared to controls. Further, a strong positive correlation was seen between serum ADA level and fasting plasma glucose level which could help in the pathogenesis of nonobese T2DM subjects.

However, there are few limitations in our study which includes the non-estimation of serum transaminase and serum insulin levels which are known to be related to ADA. Moreover, a correlation study between serum ADA level and oral glucose tolerance test will further enhance the serum level of ADA in nonobese T2DM subjects. Prediabetic nonobese subjects were also not considered in this study as screening of serum ADA may be an alarming factor in the pathogenesis of nonobese T2DM subjects.

Despite these limitations, our study shows higher serum ADA, TG, and FPG levels in nonobese T2DM patients and a strong correlation between ADA and FPG which suggests an association between ADA and nonobese T2DM subjects. A larger cross-sectional study needs to be done to conclude the fact.

## Figures and Tables

**Table 1:** Demographic and biochemical characteristics of the subjects.

	Control (*n* = 45)	T2DM (*n* = 56)
Age (in years)	52.42 ± 5.79	54.52 ± 10.66
Sex (M/F)	23/22	30/26
BMI (kg/m^2^)	24.15 ± 1.47	22.47 ± 1.79*
FPG (mg/dL)	80.91 ± 8.55	167.4 ± 60.3*
HbA_1_C (%)	4.82 ± 0.43	7.48 ± 1.08*
Serum total CHL (mg/dL)	151.3 ± 19.65	168.2 ± 28.01**
Serum TG (mg/dL)	112.4 ± 19.81	197.1 ± 81.04*
Serum HDL (mg/dL)	43.65 ± 3.65	36.82 ± 2.93*

FPG: fasting plasma glucose; CHL: cholesterol; TG: triacylglycerol; HDL: high density lipoprotein cholesterol. Age, BMI, HbA_1_C scores, and serum levels of biochemical parameters were expressed as the means ± SD. Statistically significant, *P < 0.0001; **P < 0.005.
